# Coadministration of tacrolimus with corticosteroid accelerates recovery in refractory patients with polymyositis/ dermatomyositis: a retrospective study

**DOI:** 10.1186/1471-2474-13-228

**Published:** 2012-11-22

**Authors:** Yasuhiro Shimojima, Wataru Ishii, Masayuki Matsuda, Ko-ichi Tazawa, Shu-ichi Ikeda

**Affiliations:** 1 Department of Medicine (Neurology and Rheumatology), Shinshu University School of Medicine, Matsumoto, Japan

**Keywords:** Dermatomyositis, Polymyositis, Tacrolimus

## Abstract

**Background:**

To investigate whether or not coadministration of tacrolimus (TAC) with prednisolone (PSL) can produce a beneficial effect in the treatment of polymyositis/ dermatomyositis (PM/DM).

**Methods:**

We reviewed medical records of 32 PM/DM patients who had been admitted to our hospital, and abstracted those who had received TAC in addition to oral PSL for treatment. The clinical usefulness of TAC in PM/DM was objectively evaluated focusing upon the manual muscle strength test (MMT) score, serum creatine kinase (CK) and tapering of PSL.

**Results:**

Nine patients with PM and 6 with DM were enrolled in this study. TAC was added because of difficulty in reduction of PSL in 12 patients and recurrence with corticosteroid-induced complications in the remaining 3. Both PM and DM patients showed significant increases in the MMT score and significant decreases in serum CK 1 to 3 months after starting TAC compared with before. Skin symptoms in a clinically amyopathic DM patient also improved 1 month after starting TAC. The daily dosage of PSL could be significantly reduced in both PM and DM after starting TAC compared with before. No serious adverse events ascribable to TAC occurred in any patients.

**Conclusion:**

Additional use of TAC with PSL may safely promote improvement of PM/DM and also accelerate tapering of the latter.

## Background

Polymyositis (PM) and dermatomyositis (DM) are inflammatory systemic disorders characterized clinically by involvement of skeletal muscles, such as limb weakness and myalgia, with or without skin symptoms [1]. As an autoimmune mechanism is considered to be central to the pathogenesis of these diseases, corticosteroid has usually been employed as the first-line treatment. In some patients, however, clinical symptoms are resistant to corticosteroid, or this drug is not usable at a high dose for a long period because of complications, including infection and progressive osteoporosis, particularly in elderly people. In such cases coadministration of immunosuppressive agents is the next candidate therapy.

Tacrolimus (TAC) is a relatively new immunosuppressant, which acts as a calcineurin inhibitor, and selectively and reversibly suppresses T lymphocytes secreting cytokines, such as interleukin-2 [2]. Our previous report has demonstrated that TAC can promote recovery of refractory cases of myasthenia gravis, which is one of the organ-specific autoimmune disorders, in cooperation with oral prednisolone (PSL) [3]. As activated T lymphocytes play an important role in the pathogenesis of PM/DM [4-7], coadministration of TAC with oral PSL may be able to promote early recovery from these disorders, and reduce corticosteroid-induced adverse events [4,8-11]. In the present study we retrospectively investigated the clinical course of PM/DM patients who had received TAC in addition to PSL in order to clarify the therapeutic and adverse effects of the former. We postulate that additional use of TAC may be beneficial for PM/DM patients with regard to its ability to promote early recovery and rapid dose-reduction of corticosteroid.

## Methods

### Patients

We reviewed medical records of 32 Japanese patients (11 men and 21 women; age range, 22 to 81 years; mean, 54.5 ± 9.1 years) who had been admitted to Shinshu University Hospital for treatment of PM/DM between January 2006 and August 2010. Three of the authors (YS, MM and KT) abstracted the data for serum creatine kinase (CK), visible rash, and histopathology of the muscle and/or skin before treatment, and confirmed that all of the patients suffered from PM/DM according to the criteria proposed by Bohan and Peter [1]. Demographic parameters, including age and gender, and medical history of treatment for PM/DM were abstracted simultaneously. All of the patients who had received TAC in addition to PSL were enrolled in this study. We obtained further clinical information from their medical records, such as serum CK, muscle strength, skin symptoms and the daily dosage of TAC and PSL during treatment, onset age, disease duration, autoantibodies and the history of associated diseases, particularly malignancy and interstitial pneumonia. The Committee for Medical Ethics of Shinshu University School of Medicine approved this study (No. 1986).

### Protocol for TAC administration

To check renal and liver function prior to commencement of TAC, each patient receives routine laboratory examinations, including hematology, blood chemistry, creatinine clearance and urinalysis. We administer oral TAC (Prograf®, Asteras, Osaka, Japan) to the patients with their informed consent unless laboratory data show 1.5 mg/dL or higher levels of serum creatinine or the presence of serious associated diseases, such as severe diabetes mellitus. The dosing protocol for TAC in our hospital is as follows: the daily dosage is started in principle at 2 mg in a single dose, and is increased to 3 mg after 2 weeks as long as there have been no adverse events ascribable to this drug, such as eruptions or a rapid increase in serum creatinine. We check the trough concentration of TAC at least every other month. When the trough concentration either exceeds 15 ng/mL or is between 10 and 15 ng/mL on two successive occasions, the daily dosage is reduced 1 mg at a time. On starting TAC oral PSL is continued in principle at the same dosage as before. To detect development of hypertension and abnormalities in laboratory data such as renal dysfunction and hyperglycemia as early as possible, hematology and blood chemistry as well as blood pressure are examined at least every week during admission and every month at the outpatient clinic. If the serum creatinine levels double despite dose adjustment, TAC is discontinued. The dosage of oral PSL is reduced after starting TAC at varying doses and speeds as necessary.

### Clinical and laboratory assessment of therapeutic outcomes

We used the following information as objective clinical markers for therapeutic outcomes of TAC: the results of the manual muscle strength test (MMT), which had been performed by 4 of the authors (YS, WI, MM and KT) according to the standard method [12], values of serum CK and the daily dosage of oral PSL. On the basis of the results of MMT, muscle strength in both sides of the deltoid, biceps brachii, triceps brachii, iliopsoas, quadriceps femoris and hamstrings was semiquantitavely graded before and during treatment using the rating scale from 0 to 5 (0: no contraction, 1: visible contraction but immovable, 2: movable without gravity but immovable against it, 3: movable against gravity, 4: slight decrease, 5: normal), and the sum was defined as the MMT score with a healthy state thus being 60. Increases in the MMT score and/or decreases in serum CK indicated clinical improvement of PM/DM. In DM patients with visible rash but no muscle involvement therapeutic effects of TAC were evaluated with macroscopic findings of skin symptoms alone. We abstracted clinical information on adverse events ascribable to TAC focusing upon infection, renal and liver dysfunction and hyperglycemia.

### Statistics

Wilcoxon’s signed rank test was used in order to determine statistical differences in the MMT score, serum levels of CK and the dosage of oral PSL between before and after adding TAC. The results were expressed as mean ± standard deviation, and a p-value less than 0.05 was regarded as statistically significant. Commercially available statistics software was used for data analysis (StatView for Macintosh, Abacus Concepts, Berkeley, CA, USA).

## Results

Nine patients with PM (2 men and 7 women; mean age 48.0 ± 15.2 years, range 17 to 63) and 6 with DM (1 man and 5 women; mean age 50.7 ± 18.8 years, range 24 to 72) received TAC for treatment, and met the criteria for our review. The rest were treated with either PSL alone (n = 8) or PSL and cyclosporine A (CyA) (n = 9). Clinical profiles of the 15 patients enrolled in this study are summarized in Table 1. One of the DM patients (patient 15) was compatible with clinically amyopathic DM (CADM) with regard to showing typical skin symptoms, including heliotrope rash, Gottron’s sign and ulceration, with no or little muscle involvement [13]. No patients had either malignancy or visceral organ involvement, particularly acute interstitial pneumonia, on intensive systemic survey. The anti-nuclear and anti-Jo-1 antibodies were detected in 1 patient each with PM and DM, respectively. The reason for adding TAC was difficulty in reduction of oral PSL in 12 patients and recurrence of either muscle symptoms or CK elevation in 3. As the dosage of PSL could not be increased in the latter because of corticosteroid-induced complications, such as progressive osteoporosis and glaucoma, TAC was added for treatment. On starting TAC, disease duration, the MMT score, serum CK and the daily dosage of oral PSL were 16.6 ± 45.9 months, 49.1 ± 8.6, 1089.7 ± 925.8 IU/L and 28.8 ± 17.0 mg, respectively. No abnormalities were found in laboratory data except for increases in acute phase reactants and muscle-related enzymes, such as CK and lactate dehydrogenase. All of the patients received TAC according to our protocol except for 2 (patients 6 and 9), in whom this drug was started at 1.5 mg/day and maintained at 2 mg/day at their request. The trough concentration of TAC remained between 2.5 and 6.0 ng/mL in all of the patients. We had available data for the MMT score, serum CK, the daily dosage of oral PSL and adverse events ascribable to TAC in all of the 15 patients enrolled in this study.

**Table 1:** Clinical profiles of the patients enrolled in this study

**No.**	**Sex**	**Diagnosis**	**Age of onset**	**Auto antibodies**		**Reason for adding TAC**	**On starting TAC**					**Complications ascribable to TAC**
				**Anti-Nuclear Antibody**	**Anti-Jo-1 Antibody**		**Disease duration (months)**	**Complications**	**MMT score**	**CK (IU/L)**	**Daily dosage of PSL (mg/day)**	
1	F	PM	17	-	-	DRP	3	-	50	838	35	-
2	F	PM	37	+	-	DRP	4	-	53	775	30	-
3	F	PM	46	-	-	DRP	5	-	60	666	15	-
4	M	PM	55	-	-	DRP	5	-	60	482	15	-
6	F	PM	61	-	-	DRP	1	-	47	2241	45	-
7	F	PM	63	-	-	DRP	1	-	47	965	30	-
8	F	PM	37	-	-	Recurrence	180	Osteoporosis, multiple rib fracture	40	1809	2.5	-
9	M	PM	57	-	-	Recurrence	32	Glaucoma	60	810	4	-
10	F	DM	31	-	+	DRP	1	-	47	1665	50	-
11	M	DM	58	-	-	DRP	1	-	45	612	60	-
12	F	DM	59	-	-	DRP	1	-	35	3699	30	-
13	F	DM	60	-	-	DRP	1	-	35	3699	50	-
14	F	DM	72	-	-	Recurrence	8	Osteoporosis	35	864	20	-
15	F	CADM	24	-	-	DRP	1	-	60	74	25	-

CADM: clinically amyopathic dermatomyositis, CK: creatine kinase, DM: dermatomyositis, DRP: difficulty in reduction of prednisolone, MMT: manual muscle strength test, PSL: prednisolone, TAC: tacrolimus.

**Figure 1:**
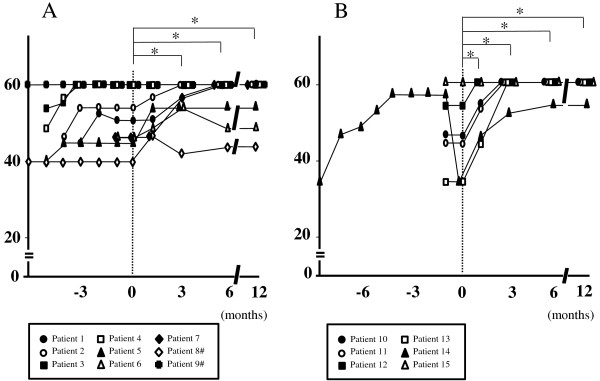
Temporal profiles of the MMT score from diagnosis in patients with PM (A) and those with DM (B), showing significant increases 3, 6 and 12 months after adding TAC compared with before (*p < 0.05). A similar significant change is seen also 1 month after adding TAC in the DM patients. Time point of adding TAC is indicated as 0. #Data of patients 8 and 9 are shown from 6 months before adding TAC.

**Figure 2:**
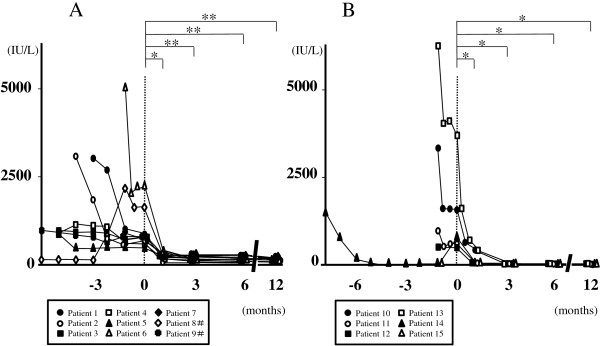
Temporal profiles of serum CK from diagnosis in patients with PM (A) and those with DM (B), showing significant decreases 1, 3, 6 and 12 months after adding TAC compared with before (*p < 0.05 and **p < 0.01). Time point of adding TAC is indicated as 0. #Data of patients 8 and 9 are shown from 6 months before adding TAC.

**Figure 3:**
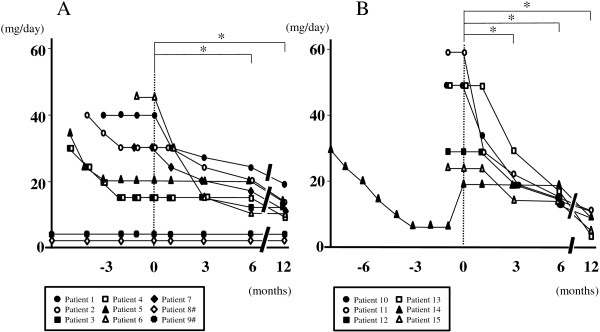
Temporal profiles of oral PSL from diagnosis in patients with PM (A) and those with DM (B), showing significant decreases 6 and 12 months after starting TAC compared with before (*p < 0.05). A similar significant change is seen also 3 months after adding TAC in the DM patients. Time point of adding TAC is indicated as 0. #Data of patients 8 and 9 are shown from 6 months before adding TAC.

Temporal profiles of the MMT score are shown in Figure 1. The MMT score was significantly higher in both PM and DM 3, 6 and 12 months after adding TAC compared with before (p < 0.05). The DM patients showed a significant increase in the MMT score also 1 month after adding TAC in comparison with before (p < 0.05). Heliotrope rash and other skin symptoms in a CADM patient persisted even with oral PSL alone, but quickly improved 1 month after adding TAC. Temporal profiles of serum CK are shown in Figure 2. In PM serum CK obviously decreased 1 month after adding TAC in all of the patients, and of these 6 showed approximate normalization in the following 2 months. No obvious reincrease in serum CK was seen in any patients. Serum CK was significantly lower 1 month after adding TAC comapred with before (p < 0.05), and a similar significant difference was seen also 3, 6 and 12 months after commencement of this drug (p < 0.01). In DM serum CK obviously decreased after adding TAC in all the patients, and was normalized in 3 months. No reincrease was seen in any patients. Serum CK was significantly lower 1, 3, 6 and 12 months after adding TAC compared with before (p < 0.05). Temporal profiles of oral PSL are shown in Figure 3. All of the patients showed successful reduction of oral PSL to 20 mg daily or less within 12 months after adding TAC. The daily dosage of PSL was significantly lower in both PM and DM 6 and 12 months after adding TAC compared with before (p < 0.05). The DM patients showed a significant decrease in the daily dosage of PSL also 3 months after adding TAC in comparison with before (p < 0.05). We could not find any clinical information or laboratory data suggestive of serious adverse events ascribable to TAC in the medical records of any patients.

## Discussion

The present study showed 2 possible advantages of using TAC in the treatment of PM/DM. One is acceleration of recovery. Both PM and DM patients showed significant increases in the MMT score and significant decreases in serum CK 1 to 3 months after starting TAC compared with before. In a patient with CADM obvious improvement of skin symptoms was seen 1 month after starting TAC. Considering that some of the clinical manifestations and laboratory data suggestive of active disease, such as muscle weakness, skin symptoms and elevated levels of serum CK, worsened or remained unimproved in all of the patients even with oral PSL alone at the same dosage for at least 1 month, commencement of TAC may have produced additional therapeutic effects on PM/DM. Few case reports have so far described such clinical efficacy of TAC in PM/DM [14-16], and the present study first demonstrated it in a series of patients. The MMT score in some PM patients fluctuated even while they were being treated with PSL and TAC, and full recovery of muscle strength was more frequently seen in DM than in PM. These results suggest that TAC may be more effective for patients with DM than for those with PM.

The other possible advantage of using TAC in the treatment of PM/DM is early and rapid reduction of corticosteroid. As oral PSL, which is often used as a kind of corticosteroid, frequently causes adverse events such as osteoporosis and compression fracture of the spine, long-term use of it should be avoided if possible, particularly in postmenopausal women. Coadministration of TAC can probably enhance immunosuppressive potential and may be able to shorten the period of high-dose corticosteroid therapy. In the present study both PM and DM patients showed significant decreases in the daily dosage of oral PSL 3 to 6 months after starting TAC compared with before. These results suggest that additional use of TAC may be able to actually reduce the daily dosage of PSL without worsening of clinical symptoms and laboratory data. PSL could be maintained at a low dose after adding TAC upon recurrence in 2 PM and 1 DM patients with serious complications ascribable to corticosteroid, such as progressive osteoporosis. These PM/DM patients also showed favorable therapeutic outcomes. Coadministration of TAC in PM/DM may be able to contribute not only to dose reduction of PSL but also to improvement of symptoms and normalization of serum CK even under low-dose PSL, particularly in cases with corticosteroid-induced complications in the long clinical course. The present study is, however, a retrospective uncontrolled one in a single center. To more clearly show the therapeutic effects of TAC in PM/DM, a randomized controlled multi-center study in a larger set of patients is necessary.

TAC sometimes causes various adverse events, such as renal dysfunction and diabetes mellitus, which depend on its blood concentration [17,18]. We, therefore, usually adjust the daily dosage of TAC based on the trough concentration in order to avoid adverse effects due to this drug. In the present study the daily dosage of TAC was 1.5 mg to 3.0 mg, and no serious adverse events occurred in any patients under a trough concentration of at most 6 ng/mL. According to recent reports, adverse effects of TAC can be adequately avoided by keeping the trough value less than 10 ng/mL [19,20]. TAC might be safely usable at or below 3 mg/day, which is officially approved as a standard dose, in PM/DM without frequent monitoring of the trough concentration as long as renal function is almost normal. We can expect potent therapeutic effects from TAC by giving the whole dose at one time as done in this study because the trough concentration is more definitively increased than when administered in a divided dose [21-23]. CyA is also an immunosuppressive agent which acts as a calcineurin inhibitor [2], but frequent adjustment of the dose according to the trough concentration is sometimes necessary in order to increase therapeutic effects and prevent adverse events. TAC might be superior to CyA with regard to the lack of need for frequent monitoring of the trough concentration, particularly when using these drugs in the outpatient clinic.

## Conclusions

Coadministration of TAC with PSL is probably the well-tolerated first-line therapy next to the latter alone in PM/DM with regard to both therapeutic effects and safety. Additional use of TAC may also lead to rapid reduction of PSL, which is particularly important for PM/DM patients with various complications ascribable to corticosteroid in the long clinical course.

## Competing interests

The authors declare that they have no competing interests.

## Authors’ contributions

YS is the main author, WI, KT and MM took part in analysis and interpretation of data, and MM and SI revised the manuscript for intellectual content. All authors read and approved the final manuscript.

## Pre-publication history

The pre-publication history for this paper can be accessed here:

http://www.biomedcentral.com/1471-2474/13/228/prepub
